# Supplementary material for: A human epithelial co-culture system reveals distinct host cell interaction behaviours for Treponema pallidum
Source: Commun Biol. 2026 Jul 7;9:1418. doi: 10.1038/s42003-026-10635-9 (PMC13720188; doi:10.1038/s42003-026-10635-9)
Supplement: Supplementary file 1 [file 42003_2026_10635_MOESM1_ESM.pdf]

nature portfolio

Peer Review File

A human epithelial co-culture system reveals distinct host cell interaction behaviours for *Treponema pallidum*

Corresponding Author: Dr Linda Grillová

This file contains all reviewer reports in order by version, followed by all author rebuttals in order by version.

Version 0:

Reviewer comments:

Reviewer #1

(Remarks to the Author)
This is a well-executed and scientifically significant study addressing a long-standing challenge in Treponema pallidum research. The authors have successfully established two human cell lines, CAL-39 (vulvar epithelial origin) and HepG2 (hepatic origin), as viable hosts for sustained in vitro T. pallidum cultivation, representing an important step toward replacing the rabbit Sf1Ep system with clinically relevant human models. Particularly noteworthy is the development of the first live-cell imaging platform for T. pallidum under microaerophilic conditions, enabling the first description of two distinct host-cell interaction behaviors: surface-associated crawling and stable single-polar attachment. These observations open genuinely new avenues for understanding spirochetal motility and tissue invasion. The manuscript is clearly written, the experimental design is logical, and the data are of high quality. I have a few suggestions that I believe could further strengthen the manuscript.

Major comments:
1. All experiments use the SS14-GFP strain, which belongs to the SS14-like genomic cluster. Since T. pallidum strains fall into two well-characterized clusters, SS14-like and Nichols-like, that differ in replication rate and potentially in host-cell interaction properties, including at least one representative Nichols-like strain in the core growth and imaging experiments would considerably broaden the manuscript's scope. This is especially relevant for the crawling and single-polar attachment behaviors, which represent one of the novel findings of the study.

2. A closely related study (Bosak et al., BMC Microbiology, 2026) independently demonstrates human cell-based T. pallidum cultivation using foreskin fibroblasts (HFF1, HFFC, and MoNa), with continuous maintenance of the DAL-1 strain for over one year. This paper does not appear to be cited in the current manuscript. I suggest adding a paragraph in the Discussion that cites Bosak et al., highlights the complementary nature of the two approaches (epithelial vs. fibroblast; mechanistic imaging vs. multi-strain breadth), and articulates what the present manuscript uniquely contributes.

3. The medium acidification observed after day 9 in CAL-39 and HepG2 co-cultures is noted as a practical limitation. This phenomenon may reflect the accumulation of metabolic by-products from actively proliferating epithelial cells over extended culture periods, rather than an inherent deficiency of the cultivation system itself. I suggest that the authors briefly discuss this in the Discussion, including potential optimization strategies for future work.

4. One of the more interesting findings of the study is the contrast between CAL-39 and SW954, both of vulvar squamous cell carcinoma origin, yet markedly different in their ability to support T. pallidum growth. The linear regression between host cell division time and T. pallidum yield ($R^2 = 0.37$, $p = 0.147$) suggests that doubling time alone cannot fully explain permissiveness. I suggest that the authors expand the Discussion to explore potential mechanisms underlying this difference, which would add meaningful depth and help frame hypotheses for future work.

Minor comment:
Lines 159–161: The analogy with Leptospira and Borrelia translocation behaviors is apt and adds useful context. Adding primary citations for the specific crawling or translocation behaviors described in each genus would help readers locate the relevant literature, given that the mechanistic basis of surface motility differs across spirochete species.

Reviewer #2

(Remarks to the Author)
Summary / overall assessment:
This well communicated manuscript explores whether human cell lines can substitute for the rabbit epithelial Sf1Ep line for Treponema pallidum co-culture under low-oxygen (1.5%) conditions. It offers a much needed and clear advance in the field. It is also laudable that the collaborative research team spans multiple continents!

The manuscript describes the clear need to move toward a human-cell–only platform, which could expand experimental options and potentially be more clinically relevant. The data suggest that two human cell lines (Cal-39 and HepG2) can sustain T. pallidum division, growth, and motility. However, the current results mainly indicate feasibility rather than demonstrating improved performance over Sf1Ep and there is need to better establish a clear clinical relevance. There are several missed opportuinties to confirm the utility of this field advancement including a more robust discussion of the differences observed for the planktonic vs adherent kinetics shown for the three supportive cultures (fig 2) and exploration of the ‘omics of both the infected mammalian cells and the 2 forms of T. pallidum that at minimum should be discussed as a value added for the novel system. A paragraph on limitations is needed in the discussion and should include the single T. pallidum isolate studied and the liquid environment that limits the recapitulation of the in vivo sites of infection. Strengthening the evidence for the manuscript’s central claims would substantially improve its impact.
• The descriptor of ‘floating’ cells should be adjusted to planktonic throughout the manuscript or explain why this descriptor was chosen. These two populations offer substantial potential for insights into the pathogenic processes engaged or disabled and this should be better addressed in the paper.
• Please state explicitly which new capabilities the human cell lines (Cal-39 and HepG2) provide in this study, versus which capabilities they enable, in principle, in future work, compared with the Sf1Ep system.
• If the manuscript positions the human cell line co-cultures as more pathogenesis- or vaccine-relevant, please discuss whether such co-cultures are expected to alter surface antigen exposure or expression (for example, Tpr proteins) in ways that change target relevance. Could T. pallidum’s capacity to maintain infectivity, virulence determinants, and antigenic variation mechanisms be altered?
• Relatedly, please clarify whether the human cell line co-culture is compatible with immune-relevant assays, such as antibody-mediated inhibition, complement sensitivity, and opsonophagocytosis using human sera, and indicate what has been tested here.
• Even if human cell lines replace Sf1Ep in the co-culture system, important translational gaps remain (tissue complexity, systemic immune responses, multi-organ dissemination). Please expand the limitations of your model, describe how they can be addressed, and compare your co-culture system to other models, such as in vitro human-cell assays, rabbit infection models, advanced ex vivo human tissues, organs on a chip, organoids, ALI, and microfluidic systems. What advantages does your model confer over them?
• Genetic manipulation efforts (e.g., KanR, tprA knockout) have been demonstrated in the Sf1Ep co-culture context, as in Ref. 4 (Romeis et al.). Can human cell substitution revalidate these approaches?
• Line 84: Please clarify whether TpCM-2 was used unchanged for human cell line co-culture, or whether any human cell-specific modifications/adaptations were required.
• Lines 108–112: Please rephrase to match what is shown in Supplementary Figure 2.
• Line 169: Please indicate whether the described motility types (including crawling) were quantified (for example: speed, directionality, attachment frequency, and persistence). If available, please comment on whether motility phenotypes correlate with viability and replication.

Reviewer #3

(Remarks to the Author)
Research on T. pallidum, the etiologic agent of syphilis, has advanced considerably by the development of a rabbit epithelial cell co-culture system by Edmondson et al. In this manuscript, Capuccini et al have addressed a significant drawback in the rabbit cell co-culture model, by screening several human epithelial cells with the aim of identifying one or more cell-lines that support T. pallidum growth to an equivalent extent as rabbit SflEp cells.

The authors have screened multiple human cell-lines, and have found two relatively slow-growing cell-lines that support bacterial growth in a co-culture model in which all other conditions are the same as the SflEp model. These two cell-lines are CAL-39, a cell-line derived from a vulval carcinoma, and HepG2, a cell-line derived from a hepatoblastoma. T. pallidum does replicate to significantly higher levels when co-cultured with CAL-39 relative to HepG2. The authors make it clear that this should not be interpreted to indicate a tropic preference for vulval-origin cells, because another vulval carcinoma cell-line, SW954, does not support T. pallidum growth/replication in their co-culture model.

The data presented indicate that T. pallidum grows as well in CAL-39 co-culture as it does in SflEp co-culture. As such, this work represents an advance over existing methods; specifically, it becomes feasible to query the role of specific human genes necessary for T. pallidum growth/replication.

The data are relatively clear and convincing.

Two issues should be addressed:

1. Figure 3C has a duplicate of the same TEM showing single-pole attachment.

2. The authors should explore the correlation between cell-division rate and support for T. pallidum co-culture in more depth. For example, human cell-lines, including HepG2 can be treated with low levels of mitomycin C for 1-2 hours, after which it can be washed out. After 24 hours, such cells accumulate at the G2/M boundary and stop cycling. Such stalled cells can be sustained in excess of 1 week. It would be useful to determine if such cells support T. pallidum growth/replication better than cycling cells? It would also be useful to know if stalling any of the non-permissive cells identified by the authors switches them to being permissive. Doing so would may permit a better correlation between cell division rate and permissivity.

Reviewer #4

(Remarks to the Author)
I co-reviewed this manuscript with one of the reviewers who provided the listed reports. This is part of the Communications Biology initiative to facilitate training in peer review and to provide appropriate recognition for Early Career Researchers who co-review manuscripts.

Version 1:

Reviewer comments:

Reviewer #1

(Remarks to the Author)
I am pleased with the revisions made by the authors and have no additional comments.

Reviewer #2

(Remarks to the Author)
The revised manuscript addresses the major concerns and provides a robust advance to the field. It is now acceptable for publication.

Reviewer #4

(Remarks to the Author)
I co-reviewed this manuscript with one of the reviewers who provided the listed reports. This is part of the Communications Biology initiative to facilitate training in peer review and to provide appropriate recognition for Early Career Researchers who co-review manuscripts.

Open Access This Peer Review File is licensed under a Creative Commons Attribution 4.0 International License, which permits use, sharing, adaptation, distribution and reproduction in any medium or format, as long as you give appropriate

credit to the original author(s) and the source, provide a link to the Creative Commons license, and indicate if changes were made.
In cases where reviewers are anonymous, credit should be given to 'Anonymous Referee' and the source.
The images or other third party material in this Peer Review File are included in the article's Creative Commons license, unless indicated otherwise in a credit line to the material. If material is not included in the article's Creative Commons license and your intended use is not permitted by statutory regulation or exceeds the permitted use, you will need to obtain permission directly from the copyright holder.
To view a copy of this license, visit https://creativecommons.org/licenses/by/4.0/

Response to referees' letter

Dear Editors,

Please find below the referees' comments addressed in a point-by-point manner. The changes in the manuscript based on the referees' comments are highlighted in yellow in the manuscript file. We have attempted to address all experimental work that was feasible within the given timeframe to strengthen the manuscript, including exploring the ability of the human co-culture system to support the growth of the Nichols strain in addition to the SS14 strain, thereby confirming the utility of the co-culture system across both major, genetically distinct lineages of T. pallidum. We also extended the duration of T. pallidum maintenance to demonstrate that longer-term co-cultivation with human cell lines is feasible.

Other suggestions for experimental work, while important and currently being pursued by our group, are beyond the scope of this revision, as they require a substantial amount of time and resources. In addition, some experiments (Reviewer 3, suggestion 2) had already been performed by our group prior to the start of this project but were not included in the manuscript because the results were not reproducible (see details below).

Reviewer #1 (Remarks to the Author):

This is a well-executed and scientifically significant study addressing a long-standing challenge in Treponema pallidum research. The authors have successfully established two human cell lines, CAL-39 (vulvar epithelial origin) and HepG2 (hepatic origin), as viable hosts for sustained in vitro T. pallidum cultivation, representing an important step toward replacing the rabbit Sf1Ep system with clinically relevant human models. Particularly noteworthy is the development of the first live-cell imaging platform for T. pallidum under microaerophilic conditions, enabling the first description of two distinct host-cell interaction behaviors: surface-associated crawling and stable single-polar attachment. These observations open genuinely new avenues for understanding spirochetal motility and tissue invasion. The manuscript is clearly written, the experimental design is logical, and the data are of high quality. I have a few suggestions that I believe could further strengthen the manuscript.

Major comments:
1. All experiments use the SS14-GFP strain, which belongs to the SS14-like genomic cluster. Since T. pallidum strains fall into two well-characterized clusters, SS14-like and Nichols-like, that differ in replication rate and potentially in host-cell interaction properties, including at least one representative Nichols-like strain in the core growth and imaging experiments would considerably broaden the manuscript's scope. This is especially relevant for the crawling and single-polar attachment behaviors, which represent one of the novel findings of the study.

We have chosen SS14 strain to be our model strain for the following reasons. First, contemporary clinical isolates of *T. pallidum* are predominantly of the SS14 lineage, which is generally much more frequent than the Nichols lineage worldwide. Most genomic surveys report SS14-clade strains accounting for approximately 80–95% of current syphilis infections, whereas Nichols-clade strains are less common and more geographically restricted; therefore, SS14 more accurately represents circulating clinical infections. Second, *T. pallidum* is genetically intractable, and the generation of gain-of-function mutants remains highly challenging and in fact the GFP-producing *T. pallidum* is first of it's kind to be generated (Grillova et al, 2025). In addition, due to the long doubling time of *T. pallidum in vitro*, any selection process for engineered strains would require more than 3 months. As a result, approaches such as generating GFP-expressing Nichols strains to be used for live imaging (as we did for the SS14) are currently beyond the scope of this study.

This being said, we agree with the reviewers that it is important to demonstrate that the Nichols strain can also be maintained in the new human co-culture system. During the revision period, we therefore revived the Nichols strain from frozen stocks and assessed it's growth over several weeks in the most successful co-culture model (using CAL-39 human cells), where growth was comparable to that observed for SS14 (Supplementary figure 4, page 19). This information was also added to the discussion section.

"Although this study primarily focused on the SS14 strain, reflecting its dominance among contemporary clinical isolates, we also demonstrated that CAL-39 cells support the Nichols strain for several weeks (Supplementary Figure 4). This confirms the utility of the cell line across both major, genetically distinct lineages of *T. pallidum*.." (Lines 242-246).

Grillová L, Romeis E, Lieberman NAP, Tantalo LC, Xu LH, Molini B, et al. Bright New Resources for Syphilis Research: Genetically Encoded Fluorescent Tags for Treponema pallidum and Sf1Ep Cells. Mol Microbiol. 2024 Aug 8. doi:10.1111/mmi.15304 PubMed PMID: 39115038.

2. A closely related study (Bosak et al., BMC Microbiology, 2026) independently demonstrates human cell-based T. pallidum cultivation using foreskin fibroblasts (HFF1, HFFC, and MoNa), with continuous maintenance of the DAL-1 strain for over one year. This paper does not appear to be cited in the current manuscript. I suggest adding a paragraph in the Discussion that cites Bosak et al., highlights the complementary nature of the two approaches (epithelial vs. fibroblast; mechanistic imaging vs. multi-strain breadth), and articulates what the present manuscript uniquely contributes.

We thank the reviewer for highlighting this highly relevant concurrent study by Bosak et al.2026. We did not include this publication in the original manuscript because it was not yet available at the time of submission and no preprint was available too. As suggested, we have now added a dedicated paragraph to the Discussion section (Lines 236 - 241) citing Bosak et al. While both studies advance the development of "humanized" cultivation systems, they provide distinct contributions. Bosak et al. established a stable fibroblast-based system supporting multiple T. pallidum strains, although growth on human fibroblasts was reported to be slower than in the standard rabbit Sf1Ep system. In contrast, our study identifies human epithelial cell lines (CAL-39) that support T. pallidum growth at levels comparable to Sf1Ep cells. Moreover, our work uniquely leverages these platforms to investigate host–pathogen interactions and reveal previously uncharacterized interaction mechanisms, including crawling and polar attachment, using live-cell imaging and electron microscopy. In addition, we have introduced a growth-curve methodology for the co-culture system, enabling a more detailed characterization of growth dynamics by monitoring both adherent and planktonic bacterial populations.

3. The medium acidification observed after day 9 in CAL-39 and HepG2 co-cultures is noted as a practical limitation. This phenomenon may reflect the accumulation of metabolic by-products from actively proliferating epithelial cells over extended culture periods, rather than an inherent deficiency of the cultivation system itself. I suggest that the authors briefly discuss this in the Discussion, including potential optimization strategies for future work.

We agree with the reviewer that the change in media colour is most likely a reflection of metabolic by-products from proliferating cells, and that changing the media or using a lower concentration of the human cell inoculum would most likely resolve this. However, here, we recommend 7 days of culture as the standard passage time (Line 141-143), and days 9 and 10 were included in the growth curve experiment under standard conditions (same mammalian cell inoculum) and without changing the media, as we were also monitoring the planktonic fraction. The growth curve was designed to

understand the dynamics between the planktonic and attached fractions, and we are by no means suggesting the use of T. pallidum from acidified conditions.

4. One of the more interesting findings of the study is the contrast between CAL-39 and SW954, both of vulvar squamous cell carcinoma origin, yet markedly different in their ability to support T. pallidum growth. The linear regression between host cell division time and T. pallidum yield (R^2 = 0.37, p = 0.147) suggests that doubling time alone cannot fully explain permissiveness. I suggest that the authors expand the Discussion to explore potential mechanisms underlying this difference, which would add meaningful depth and help frame hypotheses for future work.

We thank the reviewer for this comment and agree that the difference in permissiveness between CAL-39 and SW954 is an interesting finding. We have therefore expanded the Discussion to include potential mechanisms that may underlie differences in permissiveness, including surface receptor expression, metabolic profiles, and stress-response pathways of different cell lines, all of which could influence sustained bacterial survival and replication in vitro. In addition, we also mention that media composition may play a role. (Lines 206-2010).

Minor comment:
Lines 159–161: The analogy with Leptospira and Borrelia translocation behaviors is apt and adds useful context. Adding primary citations for the specific crawling or translocation behaviors described in each genus would help readers locate the relevant literature, given that the mechanistic basis of surface motility differs across spirochete species.

We have added the relevant literature as suggested (Lines 160 - 162).

Reviewer #2 (Remarks to the Author):

Summary / overall assessment:
This well communicated manuscript explores whether human cell lines can substitute for the rabbit epithelial Sf1Ep line for Treponema pallidum co-culture under low-oxygen (1.5%) conditions. It offers a much needed and clear advance in the field. It is also laudable that the collaborative research team spans multiple continents!

The manuscript describes the clear need to move toward a human-cell–only platform, which could expand experimental options and potentially be more clinically relevant. The data suggest that two human cell lines (Cal-39 and HepG2) can sustain T. pallidum division, growth, and motility. However, the current results mainly indicate feasibility rather than demonstrating improved performance over Sf1Ep and there is need to better establish a clear clinical relevance. There are several missed opportuinties to confirm the utility of this field advancement including a more robust discussion of the differences observed for the planktonic vs adherent kinetics shown for the three supportive cultures (fig 2) and exploration of the 'omics of both the infected mammalian cells and the 2 forms of T. pallidum that at minimum should be discussed as a value added for the novel system. A paragraph on limitations is needed in the discussion and should include the single T. pallidum isolate studied and the liquid environment that limits the recapitulation of the in vivo sites of infection. Strengthening the evidence for the manuscript's central claims would substantially improve its impact.

• The descriptor of 'floating' cells should be adjusted to planktonic throughout the manuscript or explain why this descriptor was chosen. These two populations offer substantial potential for

insights into the pathogenic processes engaged or disabled and this should be better addressed in the paper

We thank the reviewer for this suggestion and agree that "planktonic" is a more appropriate descriptor than "floating" for the non-adherent *T. pallidum* population. We have therefore replaced the term "floating" with "planktonic" throughout the manuscript and in the figures.

We also agree that the attached and planktonic populations may provide important insights into *T. pallidum* pathogenic processes and host interaction dynamics. In the current study, both attached and planktonic populations were included not only to assess the ability of human cell lines to support *T. pallidum* growth and maintenance in vitro, but also because both populations are consistently observed in the established Sf1Ep co-culture system. Assessing their presence and dynamics in the human-cell co-cultures was therefore important to determine whether the newly established models recapitulate key features of the existing cultivation platform. While functional characterization of these populations is highly relevant, a detailed mechanistic investigation is beyond the scope of this manuscript, whose primary aim was to establish and characterize an alternative human epithelial co-culture system. To address the reviewer's point, we have expanded the Discussion (**Lines 223- 228**) to further acknowledge the potential biological significance of these two populations and their relevance for future studies of *T. pallidum* pathogenesis. In parallel, transcriptomic, proteomic, and metabolomic analyses comparing attached and planktonic populations are currently underway in our laboratory and will be the subject of future work.

• Please state explicitly which new capabilities the human cell lines (Cal-39 and HepG2) provide in this study, versus which capabilities they enable, in principle, in future work, compared with the Sf1Ep system.

We appreciate the need to distinguish between the capabilities directly demonstrated in this study and those enabled by the establishment of a human-cell-based co-culture platform.
In the present study, we demonstrate that the human epithelial cell lines Cal-39 and HepG2 can support sustained *T. pallidum* survival, replication, motility, attachment dynamics, and serial passage at levels comparable to the established Sf1Ep system.

Importantly, the use of human cell lines extends the utility of the co-culture system beyond bacterial propagation alone. While not directly explored in the current manuscript, human-cell-based systems provide a foundation for future mechanistic studies of host–pathogen interactions in a biologically and translationally relevant context. In particular, discoveries made using rabbit-derived systems often require subsequent validation in human models because host signalling pathways and epithelial interactions may differ substantially between species.

In addition, human co-culture systems provide important practical advantages for downstream systems biology analyses. Human transcriptomic, proteomic, metabolomic, and pathway-analysis resources are substantially more comprehensive and accessible than those available for rabbit-derived cells, facilitating integration with established bioinformatic pipelines and improving interpretation of host-response data.

To make this clearer in the manuscript, we have modified the paragraph in the introduction section (Lines 57-63) as well as in the discussion section (Lines 247-260).

• If the manuscript positions the human cell line co-cultures as more pathogenesis- or vaccine-relevant, please discuss whether such co-cultures are expected to alter surface antigen exposure or expression (for example, Tpr proteins) in ways that change target relevance. Could T. pallidum's

capacity to maintain infectivity, virulence determinants, and antigenic variation mechanisms be altered?

We agree that the interaction between host environment and *T. pallidum* antigen expression, including Tpr proteins and antigenic variation mechanisms, represents a critical area for future investigation.

Current evidence (Giacani et al. 2010) suggests that diversification of Tpr proteins such as TprK is strongly associated with immune selection pressure during infection, particularly adaptive humoral responses, rather than epithelial-cell interaction alone. Studies have demonstrated that TprK sequence diversity accumulates more rapidly in immunocompetent compared with immunosuppressed experimental animals, supporting a role for immune-driven antigenic variation in syphilis pathogenesis.

Importantly, the co-culture system described in our study does not include immune cells or other immune effectors. Therefore, although we observed distinct host-cell interaction behaviours, including crawling-like motility and stable single-polar attachment, we do not currently interpret these observations as evidence of altered surface antigen expression or modulation of antigenic variation mechanisms.

At present, our findings support the use of these human epithelial co-culture systems as models to study epithelial interaction dynamics and bacterial maintenance in a human cellular context, representing an initial step toward more clinically relevant models for T. pallidum pathogenesis. We agree that investigation of immune evasion, infectivity maintenance, and antigenic modulation will likely require more complex systems incorporating immune components, such as macrophages, serum factors, or other immune-relevant cellular models.

Giacani L, Molini BJ, Kim EY, Godornes BC, Leader BT, Tantalo LC, Centurion-Lara A, Lukehart SA. Antigenic variation in Treponema pallidum: TprK sequence diversity accumulates in response to immune pressure during experimental syphilis. J Immunol. 2010 Apr 1;184(7):3822-9. doi: 10.4049/jimmunol.0902788. Epub 2010 Feb 26. PMID: 20190145; PMCID: PMC3042355.

• Relatedly, please clarify whether the human cell line co-culture is compatible with immune-relevant assays, such as antibody-mediated inhibition, complement sensitivity, and opsonophagocytosis using human sera, and indicate what has been tested here.

We thank the reviewer for this question. To clarify, none of the specific immune-relevant assays mentioned (antibody-mediated inhibition, complement sensitivity, or opsonophagocytosis) were tested in the current study. However, in general, the co-culture system is compatible with antibody-mediated inhibition assays, although only for assessing the impact on T. pallidum viability, adherence, or motility. Because the primary objective of this study was to establish a baseline model characterizing the direct biophysical and cellular interactions between T. pallidum and human epithelial cells in an immune-isolated environment, these assays fall outside the scope of the current work.

Even if human cell lines replace Sf1Ep in the co-culture system, important translational gaps remain (tissue complexity, systemic immune responses, multi-organ dissemination). Please expand the limitations of your model, describe how they can be addressed, and compare your co-culture system to other models, such as in vitro human-cell assays, rabbit infection models, advanced ex vivo human tissues, organs on a chip, organoids, ALI, and microfluidic systems. What advantages does your model confer over them?

We thank the reviewer for this important point and agree that substantial translational gaps remain between simplified in vitro co-culture systems and the complexity of human syphilis infection in vivo.

The current model does not recapitulate tissue architecture, vascularization, multi-organ dissemination, or the systemic immune responses associated with syphilis infection. In addition, the co-culture system uses immortalized epithelial monolayers and liquid culture conditions that cannot fully reproduce the complexity of human mucosal or tissue environments.

We agree that advanced experimental systems may ultimately provide more physiologically relevant environments for studying T. pallidum pathogenesis. However, many of these systems are technically demanding, costly, difficult to standardize, or currently incompatible with the delicate culture conditions required for long-term T. pallidum maintenance.

The human epithelial co-culture system presented here serves as a foundational and experimentally practical platform that bridges the gap between the rabbit-derived Sf1Ep system and future, higher-complexity human infection models. The major advantages of this system include long-term bacterial maintenance, experimental accessibility, compatibility with live-cell imaging and molecular analyses, reproducibility across laboratories, and integration with widely available human-specific systems biology resources.

We have added a paragraph addressing these points to the Discussion section (Lines 247–259).

• Genetic manipulation efforts (e.g., KanR, tprA knockout) have been demonstrated in the Sf1Ep co-culture context, as in Ref. 4 (Romeis et al.). Can human cell substitution revalidate these approaches?

The genetic manipulation approaches described by Romeis et al., including KanR and tprA knockout generation, target *T. pallidum* directly rather than the mammalian co-culture cell line. Therefore, based on the comparable ability of Cal-39 and HepG2 cells to support sustained *T. pallidum* growth, motility, and serial passage, we do not currently anticipate that substitution of Sf1Ep cells with human epithelial cell lines would fundamentally prevent application of these genetic manipulation workflows.

• Line 84: Please clarify whether TpCM-2 was used unchanged for human cell line co-culture, or whether any human cell-specific modifications/adaptations were required.

We thank the reviewer for requesting this clarification. TpCM-2 medium was used unchanged for all experiments involving human cell line co-cultures, and no cell line–specific modifications or adaptations were introduced. We have clarified this explicitly in the Methods section (**Line 274-275**).

• Lines 108–112: Please rephrase to match what is shown in Supplementary Figure 2.

We thank the reviewer for this comment. We have revised the text (**Lines 108-112**) to ensure that the description of statistical comparisons accurately reflects the pairwise structure of the Tukey HSD analysis presented in Supplementary Fig. 2.

• Line 169: Please indicate whether the described motility types (including crawling) were quantified (for example: speed, directionality, attachment frequency, and persistence). If available, please comment on whether motility phenotypes correlate with viability and replication.

We appreciate the reviewer's suggestion. Motility phenotypes, including crawling-like movement and single-polar attachment, were assessed qualitatively by live-cell imaging and microscopy rather than through formal quantitative tracking analyses. The quantitative analyses presented in the manuscript

are limited to overall *T. pallidum* counts, motile fraction estimates, and the relative proportions of adherent versus planktonic populations across conditions. We have clarified this in the methods section (**Lines 318-320**) of the revised manuscript to better reflect the scope of the analysis.

Reviewer #3 (Remarks to the Author):

Research on T. pallidum, the etiologic agent of syphilis, has advanced considerably by the development of a rabbit epithelial cell co-culture system by Edmondson et al. In this manuscript, Capuccini et al have addressed a significant drawback in the rabbit cell co-culture model, by screening several human epithelial cells with the aim of identifying one or more cell-lines that support T. pallidum growth to an equivalent extent as rabbit SflEp cells.

The authors have screened multiple human cell-lines, and have found two relatively slow-growing cell-lines that support bacterial growth in a co-culture model in which all other conditions are the same as the SflEp model. These two cell-lines are CAL-39, a cell-line derived from a vulval carcinoma, and HepG2, a cell-line derived from a hepatoblastoma. T. pallidum does replicate to significantly higher levels when co-cultured with CAL-39 relative to HepG2. The authors make it clear that this should not be interpreted to indicate a tropic preference for vulval-origin cells, because another vulval carcinoma cell-line, SW954, does not support T. pallidum growth/replication in their co-culture model.

The data presented indicate that T. pallidum grows as well in CAL-39 co-culture as it does in SflEp co-culture. As such, this work represents an advance over existing methods; specifically, it becomes feasible to query the role of specific human genes necessary for T. pallidum growth/replication.

The data are relatively clear and convincing. Two issues should be addressed:

1. Figure 3C has a duplicate of the same TEM showing single-pole attachment.

Thanks for this comment. I hope this is visible in all versions of the manuscript, but while the TEM figures shown in Figure 3C are indeed identical, we used the second copy to visualize where the *T. pallidum* cell is located, as well as to add stars and arrows indicating the possible attachment points. To make this clearer, we have added the following sentence to the Figure 3 legend: “The red line indicates the localization of *T. pallidum* cell, and the stars and arrows indicate possible attachment sites.” (Lines 380-381).

2. The authors should explore the correlation between cell-division rate and support for T. pallidum co-culture in more depth. For example, human cell-lines, including HepG2 can be treated with low levels of mitomycin C for 1-2 hours, after which it can be washed out. After 24 hours, such cells accumulate at the G2/M boundary and stop cycling. Such stalled cells can be sustained in excess of 1 week. It would be useful to determine if such cells support T. pallidum growth/replication better than cycling cells? It would also be useful to know if stalling any of the non-permissive cells identified by the authors switches them to being permissive. Doing so would may permit a better correlation between cell division rate and permissivity.

We agree that this is an interesting and important point. In our preliminary experiments performed before this project, we observed that *T. pallidum* growth appeared better supported in fast-growing HEK293 cells when proliferation was inhibited using palbociclib, suggesting that reduced cell-cycle activity, while maintaining metabolic function, may enhance bacterial maintenance. In contrast, untreated HEK293 cells did not support sustained *T. pallidum* growth. However, when extending this approach to the co-culture systems described in this study, we were not able to reproduce these

findings consistently, and the biological replicates in such an unstable system were too variable and insufficient to draw robust conclusions, which was one of the reasons why these data were not included in this manuscript.

More importantly, pharmacological cell-cycle arrest also perturbs the overall physiology and dynamics of the co-culture system, making it difficult to disentangle effects on bacterial maintenance from broader changes in host-cell state. It also compromises the primary aim of our model, which is to establish stable, physiologically relevant human epithelial interactions that can be monitored over time.

“Importantly, although human cell division time may contribute to cell permissiveness, regression analysis in this study showed only a positive but non-significant association (Figure 1C). This suggests it is unlikely to be the primary determinant, and that other factors, such as media composition, surface receptor expression, metabolic profiles or stress-response pathways, may also play a role” (Lines 206-2010).

Reviewer #4 (Remarks to the Author):

I co-reviewed this manuscript with one of the reviewers who provided the listed reports. This is part of the Communications Biology initiative to facilitate training in peer review and to provide appropriate recognition for Early Career Researchers who co-review manuscripts.
