# Supplementary material for: A human epithelial co-culture system reveals distinct host cell interaction behaviours for Treponema pallidum
Source: Commun Biol. 2026 Jul 7;9:1418. doi: 10.1038/s42003-026-10635-9 (PMC13720188; doi:10.1038/s42003-026-10635-9)
Supplement: Supplementary file 2 [file 42003_2026_10635_MOESM2_ESM.pdf]

Supplementary material

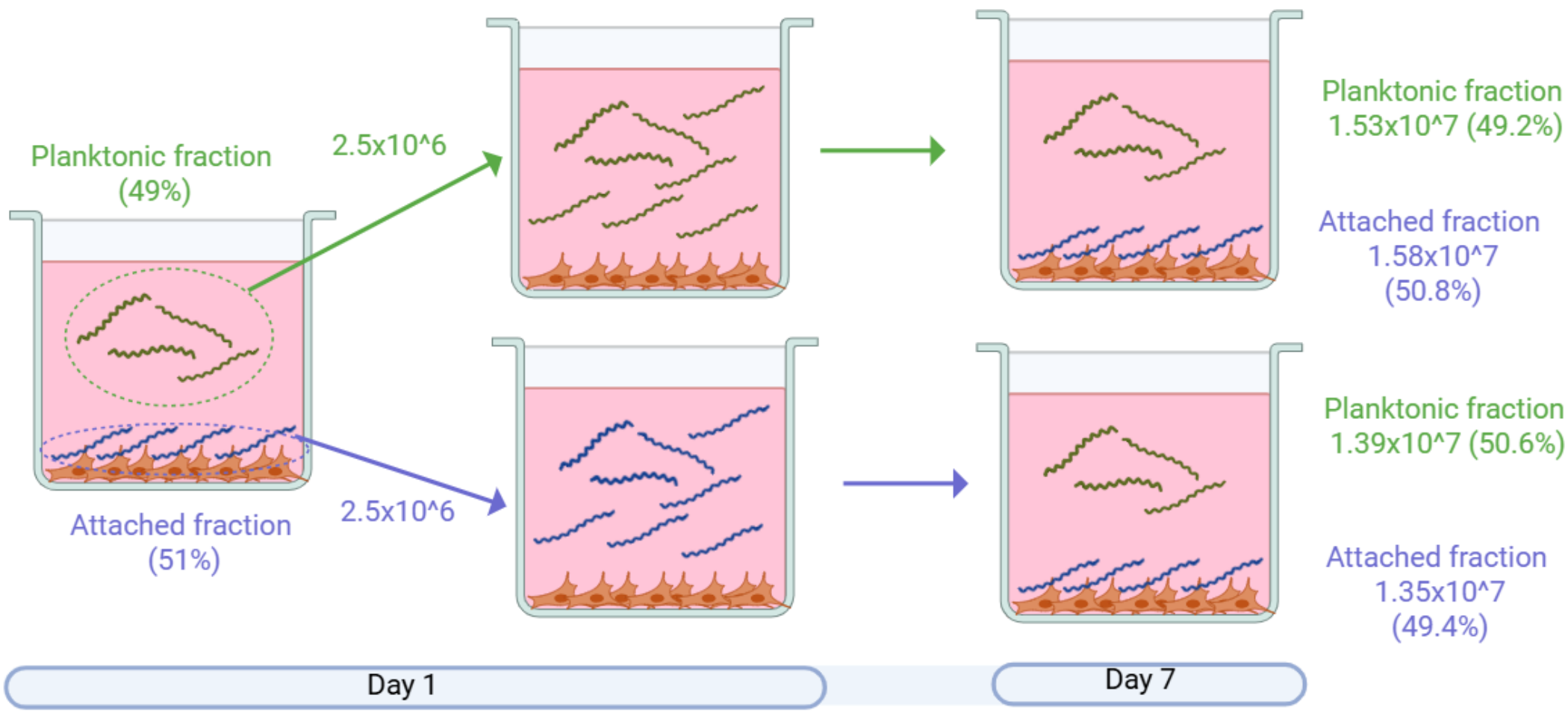

Supplementary Figure 1. Re-seeding demonstrates the replicative competence of planktonic *T. pallidum* cells. Schematic representation of re-seeding experiments demonstrating that *T. pallidum* cells recovered exclusively from culture supernatant can reattach to fresh host cell monolayers and complete a full replication cycle. Equal numbers of *T. pallidum* (2.5×10^6), either collected from the planktonic fraction (culture medium only) or from the attached fraction (host cell–associated bacteria released by trypsinization), were inoculated into fresh media and monolayers. After 7 days, both conditions yielded comparable total bacterial counts and similar proportions of planktonic and host cell–associated fractions. These results demonstrate that planktonic *T. pallidum* remain competent for reattachment and replication. Values represent mean counts and percentages from three independent biological replicates. Created in BioRender. Grillova, L. (2026) https://BioRender.com/h0yug8y

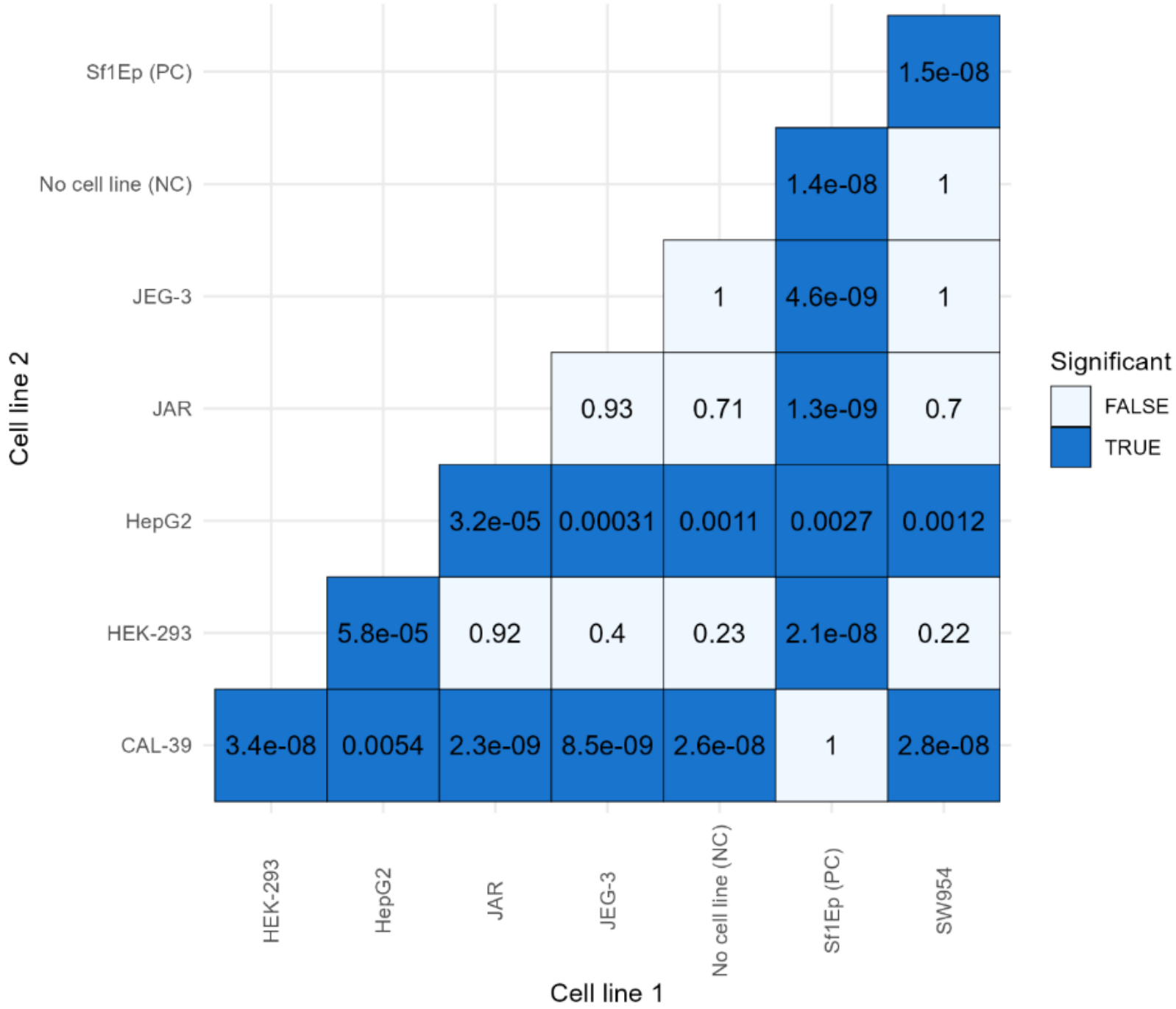

Supplementary Figure 2. Triangular heatmap of pairwise TukeyHSD post hoc comparisons of *T. pallidum* counts between cell lines. Each cell line shows the adjusted p-value for a given pairwise comparison; blue tiles indicate statistically significant differences ($p < 0.05$).

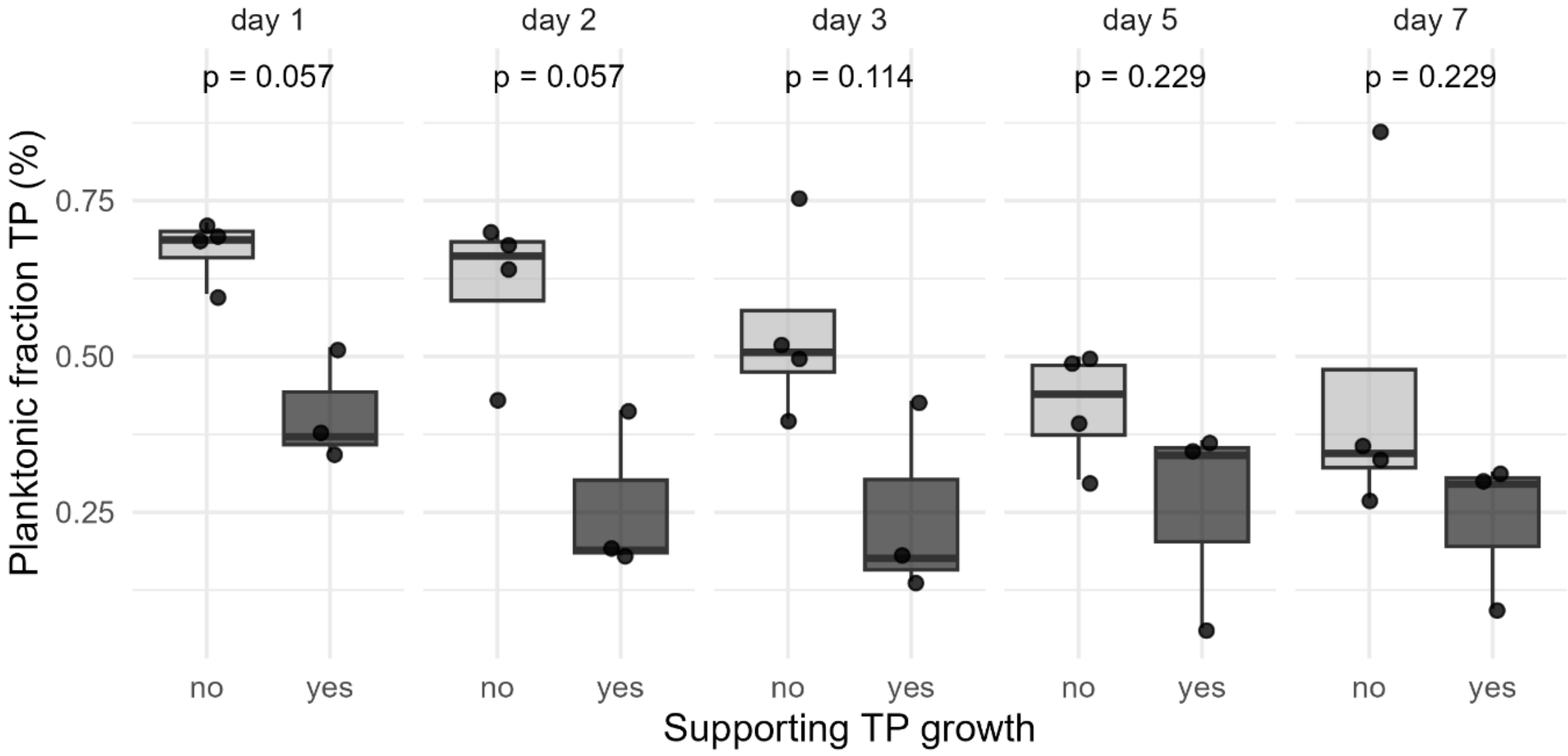

Supplementary Figure 3. Comparison of Planktonic *T. pallidum* Populations in Supporting and Non-Supporting Host Cell Lines. Planktonic fraction of *T. pallidum* in supporting (Sf1Ep, CAL-39, HEPG2) and non-supporting (HEK-293, SW954, JAR, JEG-3) cell lines across days 1, 2, 3, 5, and 7. Boxplots show the distribution of planktonic *T. pallidum* fractions for each group, with individual data points overlaid. Wilcoxon rank-sum tests were performed separately for each day (day 1, p = 0.057; day 2, p = 0.057; day 3, p = 0.114; day 5, p = 0.229; day 7, p = 0.229).

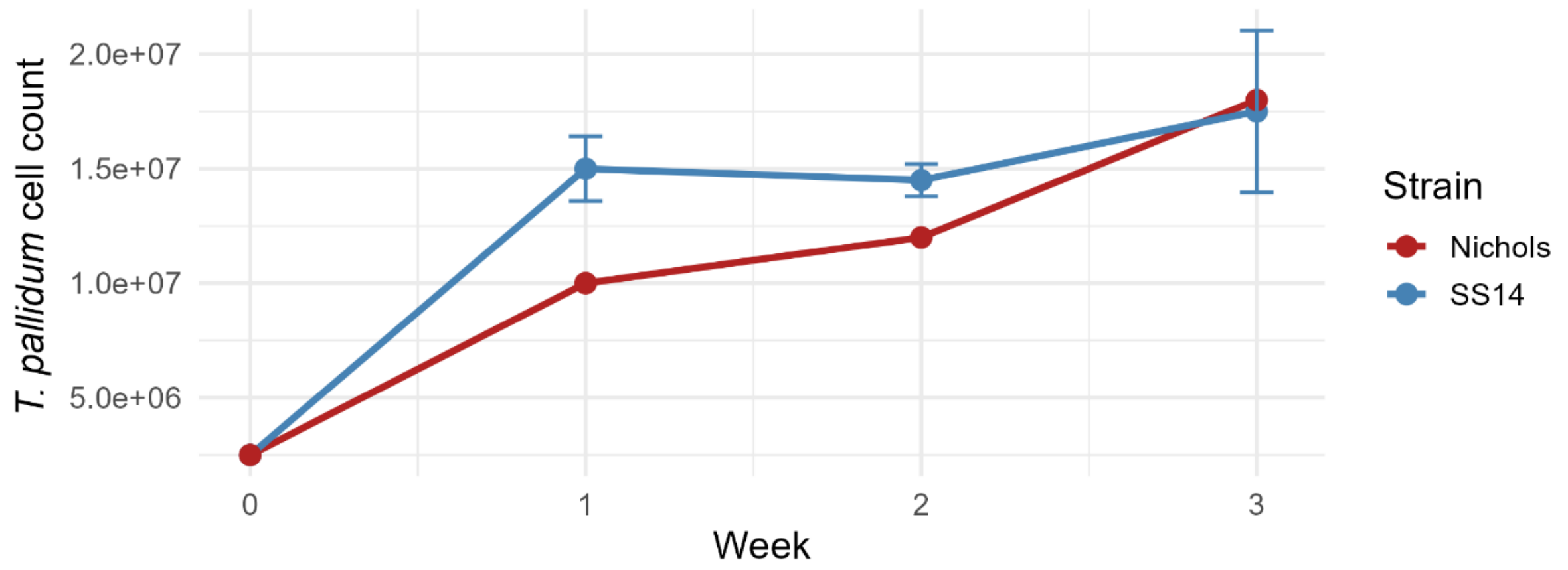

Supplementary Figure 4: Growth of *T. pallidum* SS14 and Nichols in the presence of CAL-39. A linear mixed-effects model showed no significant strain × week interaction ($F = 0.0076$), indicating no detectable differences in growth trends between SS14 and Nichols.

Supplementary Table 1. Pairwise contrasts from a linear model comparing CAL-39, HepG2, and Sf1Ep across 10 days. Reported are estimated differences, standard errors, degrees of freedom, t-ratios, adjusted p-values. Significant differences after adjustment were observed primarily at later time points, including CAL-39 vs HepG2 on days 6 and 8, and contrasts involving Sf1EP on days 9 and 10, indicating time-dependent divergence between cell lines.

Contrast	Day	Difference	Se	Df	T_Ratio	P_Adjusted
**CAL-39 - HEPG2**	day_1	3746000	5254010	58	0.712979	0.756833
**CAL-39 - SF1EP**	day_1	2313333	5254010	58	0.440299	0.898848
**HEPG2 - SF1EP**	day_1	-1432667	5254010	58	-0.27268	0.959869
**CAL-39 - HEPG2**	day_2	9020667	5254010	58	1.716911	0.207578
**CAL-39 - SF1EP**	day_2	6734000	5254010	58	1.281688	0.411135
**HEPG2 - SF1EP**	day_2	-2286667	5254010	58	-0.43522	0.901043
**CAL-39 - HEPG2**	day_3	10901333	5254010	58	2.07486	0.104008
**CAL-39 - SF1EP**	day_3	7410000	5254010	58	1.410351	0.342288
**HEPG2 - SF1EP**	day_3	-3491333	5254010	58	-0.66451	0.784895
**CAL-39 - HEPG2**	day_4	8213333	5254010	58	1.563251	0.269663
**CAL-39 - SF1EP**	day_4	5490000	5254010	58	1.044916	0.551868
**HEPG2 - SF1EP**	day_4	-2723333	5254010	58	-0.51833	0.862722
**CAL-39 - HEPG2**	day_5	6898667	5254010	58	1.313029	0.393771
**CAL-39 - SF1EP**	day_5	3283333	5254010	58	0.62492	0.80708
**HEPG2 - SF1EP**	day_5	-3615333	5254010	58	-0.68811	0.771345
**CAL-39 - HEPG2**	day_6	13632667	5254010	58	2.594717	0.031641
**CAL-39 - SF1EP**	day_6	2283333	5254010	58	0.434589	0.901316
**HEPG2 - SF1EP**	day_6	-1.1E+07	5254010	58	-2.16013	0.086819
**CAL-39 - HEPG2**	day_7	11850000	5254010	58	2.25542	0.070455
**CAL-39 - SF1EP**	day_7	2673333	5254010	58	0.508818	0.867355
**HEPG2 - SF1EP**	day_7	-9176667	5254010	58	-1.7466	0.196854
**CAL-39 - HEPG2**	day_8	16310000	5254010	58	3.104296	0.00817
**CAL-39 - SF1EP**	day_8	4830000	5254010	58	0.919298	0.630397
**HEPG2 - SF1EP**	day_8	-1.1E+07	5254010	58	-2.185	0.082271
**CAL-39 - HEPG2**	day_9	5723333	5254010	58	1.089327	0.524476
**CAL-39 - SF1EP**	day_9	-1.7E+07	5254010	58	-3.32698	0.004297
**HEPG2 - SF1EP**	day_9	-2.3E+07	5254010	58	-4.41631	0.00013
**CAL-39 - HEPG2**	day_10	2703333	5254010	58	0.514528	0.864582
**CAL-39 - SF1EP**	day_10	-2.8E+07	5254010	58	-5.41935	3.58E-06
**HEPG2 - SF1EP**	day_10	-3.1E+07	5254010	58	-5.93388	5.22E-07
